# *Legionella* Lem26 functions as an ATG8-activated effector that inhibits host autophagy

**DOI:** 10.1128/mbio.03595-25

**Published:** 2026-02-05

**Authors:** Kevin R. Parducho, Zi Yang, Emily Guinn, Daniel Choi, Shanta Nag, Thomas J. Melia, Craig R. Roy

**Affiliations:** 1 Department of Microbial Pathogenesis, Yale University School of Medicine 198940 , New Haven, Connecticut, USA; 2 Department of Cell Biology, Yale University School of Medicine 198944 , New Haven, Connecticut, USA; University of California, Berkeley, Berkeley, California, USA

**Keywords:** type IV secretion, autophagy, bacterial effectors, ADP ribosylation

## Abstract

The intracellular pathogen *Legionella pneumophila* has evolved multiple effector proteins delivered into host cells by the Dot/Icm Type IVb secretion system that prevents recognition of the vacuole in which it resides by the host autophagy pathway. The number of effectors involved in this process remains unclear. Thus, we conducted a screen in *Saccharomyces cerevisiae* to identify *Legionella* effectors that were sufficient to block autophagy. This screen identified the *Legionella* protein Lem26 as an effector capable of autophagy inhibition. Lem26 production inhibited the recruitment of core autophagy proteins to autophagic targets and prevented the proteolytic processing of autophagy substrates in both yeast and mammalian systems. The Lem26 protein encodes an ADP-ribosyltransferase (ART) domain that was found to be essential for anti-autophagy activity. *In vitro* studies showed that purified Lem26 was inactive in solution, but the addition of pre-autophagosomal membranes obtained from fractionated mammalian cell lysates stimulated Lem26 ART activity. The addition of synthetic membranes containing lipid-conjugated ATG8 proteins was sufficient to stimulate Lem26 activity *in vitro*. An ATG8-interacting motif identified in Lem26 was critical for the activation of Lem26. These data establish that Lem26 is a *Legionella* effector that is recruited and activated upon interaction with autophagic membranes, and this promotes the posttranslational modification of proteins on the autophagic membrane to arrest the autophagy pathway.

**IMPORTANCE:**

Bacterial pathogens have evolved intricate mechanisms to specifically avoid detection by the host autophagy pathway, which is a cell-autonomous innate immune pathway conserved in all eukaryotic organisms. The intracellular pathogen *Legionella pneumophila* has co-evolved with evolutionarily diverse protozoan hosts for over 100 million years. Thus, these bacteria have devised multiple strategies for evading host autophagy. In this study, we analyzed roughly 300 different *Legionella* effector proteins for their ability to disrupt autophagy in yeast. The *Legionella* effector protein Lem26 was found to specifically block autophagy in both yeast and mammalian cells. Biochemical studies revealed that this protein is tightly regulated and is activated upon binding to autophagosomal membranes, which stimulates Lem26 ADP-ribosyltransferase activity and results in the modification of critical autophagy proteins colocalized to these membranes. Thus, Lem26 has evolved the capacity to disrupt host autophagy by proximity labeling of host determinants on autophagosomal membranes, which represents a unique strategy for autophagy inhibition.

## INTRODUCTION

*Legionella pneumophila* is an intracellular bacterial pathogen that targets evolutionarily conserved eukaryotic membrane transport pathways to create the *Legionella*-containing vacuole (LCV) inside phagocytes that supports intracellular replication (1, 2). Manipulation of host membrane transport requires a type IVb secretion system called Dot/Icm that delivers proteins called “effectors” into the host cytoplasm (3, 4). These effectors promote interactions between the LCV and host ER-derived vesicles (5, 6), disrupt degradative pathways by preventing the fusion of the bacterial phagosome with endosomal vesicles (7), and interfere with the biogenesis of autophagosomes (8).

Autophagy is one of the conserved host pathways that is manipulated by *Legionella* (1). Autophagy is a stress-response pathway that sequesters diverse substrates that include protein aggregates, damaged organelles, and invading pathogens into an autophagosome that fuses with lysosomes, leading to the destruction and turnover of the substrates (9). This process, which requires the carefully orchestrated function of >20 conserved autophagy-related proteins (Atg/ATG), can be divided into distinct stages (10, 11). The process of initiation involves the Atg1/ULK1 kinase complex responsible for the phosphorylation and activation of downstream autophagy proteins (12). The nucleation stage involves the recruitment of pre-autophagosomal ATG9 vesicles (13) and production of phosphatidyl-inositol-3-phosphate (PI3P) by the phosphoinositide 3-kinase complex I (PI3KC1) (14). Expansion of pre-autophagosomal membranes involves the lipid transfer (15, 16) and lipidation complexes (17), which generates a cup-shaped structure with a highly curved rim and promotes the covalent attachment of ubiquitin-like Atg8 family proteins to the phospholipid membrane, respectively. The closure step transforms the cup-shaped phagophore into a double-membraned vesicle called the autophagosome that encapsulates cytosolic substrates in the lumen of the vesicle (18). Finally, the mature autophagosome will then fuse with lysosomes to initiate degradation of substrates. The importance of autophagy both as a quality control mechanism as well as a form of cell-autonomous defense is evidenced by its high degree of conservation in eukaryotic systems (from yeast to mammalian cells) as well as its ability to interfere with the replication of several pathogens (19–21).

*Legionella*, which evolved redundant mechanisms to survive within protozoan hosts in nature, disrupts autophagy at multiple stages using effectors that have unique biochemical functions and targets (8, 22–26). For example, the SidE family of *Legionella* effectors post-translationally modify ubiquitin to disrupt the process by which autophagy adapters can bind to ubiquitin on the LCV (24–26). The effector Lpg1137 is a serine protease that cleaves syntaxin-17 (23), which participates in the recruitment of ATG14 and PI3KC1 as well as fusion of mature autophagosomes with endolysosomal organelles (27). The effector RavZ is a cysteine protease that deconjugates ATG8 proteins on autophagosomal membranes to globally disrupt autophagy during infection (8, 28). Importantly, *Legionella* mutants deficient in combinations of these effectors still retain the ability to evade autophagic detection, which indicates there are likely other, undiscovered effectors encoded by *Legionella* capable of targeting this host pathway.

Utilizing the high degree of conservation of autophagy proteins in diverse eukaryotic organisms, we screened a library of *Legionella* proteins in *Saccharomyces cerevisiae* to identify additional effectors that interfere with autophagy.

## RESULTS

### Identification of Lem26 as an anti-autophagy effector

**Fig 1:**
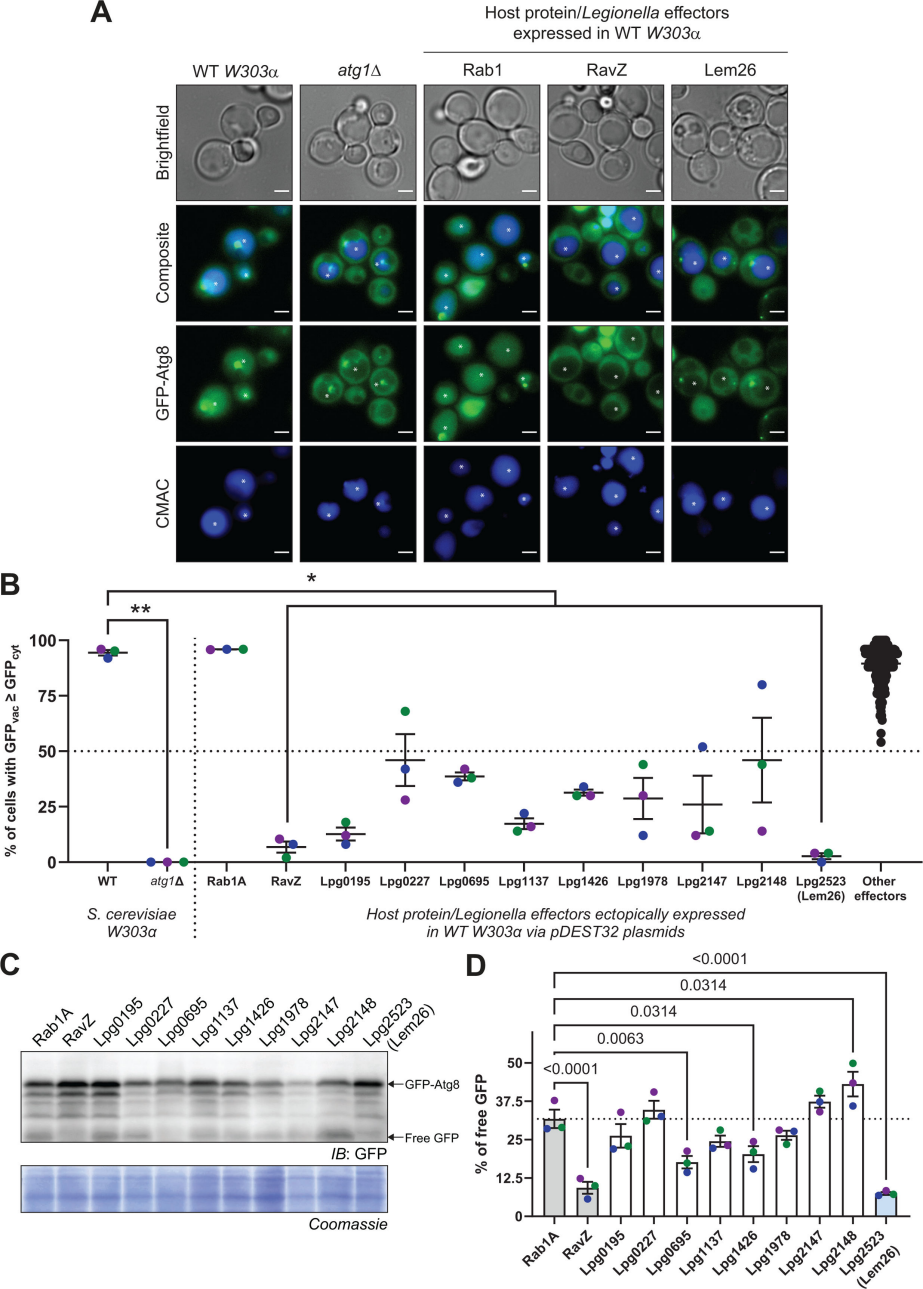
Primary and secondary screens assessing defects in vacuolar traffic and processing of GFP-Atg8 identify Lem26 (Lpg2523) as an autophagy-inhibiting protein. (**A**) Representative images of *S. cerevisiae* expressing endogenously tagged GFP-Atg8 ± plasmid-encoded effectors exposed to 200 ng/μL of rapamycin and 1 μM 7-aminochloromethylcoumarin (CMAC) (vacuolar dye) for 4 h. Vacuoles are marked with an asterisk (*). Scale bar = 2.5 μm. (**B**) Quantification of GFP-Atg8 translocation to the vacuole. Individual cells were qualitatively scored in a binary fashion, receiving a positive score if the vacuolar GFP signal was qualitatively greater than or equal to the cytosolic signal. Data points represent the percent of cells with a positive vacuolar signal from *n* = 3 independent experiments, with error bars representing means ± SEM. Results from other tested effectors that did not result in significant (>50% reduction) or reproducible phenotypes were pooled under “other tested effectors.” Statistical significance was determined using the Kruskal-Wallis with Dunn’s multiple comparison test, with each sample compared to wild-type (WT) *W303α*. *P < 0.05; **P < 0.005. (**C**) Representative immunoblot for GFP-Atg8 processing in *S. cerevisiae* expressing plasmid-encoded GFP-Atg8 ± encoded effectors. Yeast cells were grown to log phase in standard C-LEU medium prior to the addition of 200 ng/μL of rapamycin for 4 h and collection of cell pellets. (**D**) Quantification of the relative amounts of free GFP relative to the total GFP signal (free GFP + full-length GFP-Atg8) in yeast expressing Rab1A or each individual effector. Data points represent the percentage of free GFP from *n* = 3 independent experiments, with bars representing means ± SEM. Statistical significance was determined using ordinary one-way ANOVA with Holm-Sidak’s multiple comparisons test. Exact *P*-values are shown.

To identify additional anti-autophagy proteins, a library of *Legionella* effectors constructed previously (29) was screened by producing individual effector proteins in a yeast strain encoding GFP-Atg8 (Fig. 1A and B). *Legionella* anti-autophagy effectors should prevent delivery of GFP-Atg8 to the yeast vacuole, which is a process that requires a functional autophagy pathway. RavZ, which was previously identified as an autophagy-inhibiting effector (8), was used as a positive control in this screen. Yeast producing RavZ displayed a diffuse and cytosolic GFP-ATG8 signal, which contrasted with the GFP-ATG8 signal enriched in the vacuoles of the parental strain (30). Yeast producing the *Legionella* effector Lem26 displayed a diffuse cytosolic GFP-ATG8 signal that was similar to that observed in yeast producing RavZ, suggesting Lem26 could have a potent anti-autophagy activity (Fig. 1A and B). As a secondary screen, cell lysates from yeast expressing effectors that consistently displayed reduced transport of GFP-Atg8 to the vacuole were probed by immunoblot analysis to measure the proteolytic cleavage of GFP-Atg8, which occurs upon delivery of this chimeric protein to the vacuole (Fig. 1C). Quantification of GFP-Atg8 processing indicated that production of Lpg0695, Lpg1426, and Lem26 each inhibited vacuolar processing of GFP-Atg8, with the most robust phenotype being observed in yeast producing Lem26 (Fig. 1D).

### Lem26 disrupts host autophagy in mammalian cells

**Fig 2:**
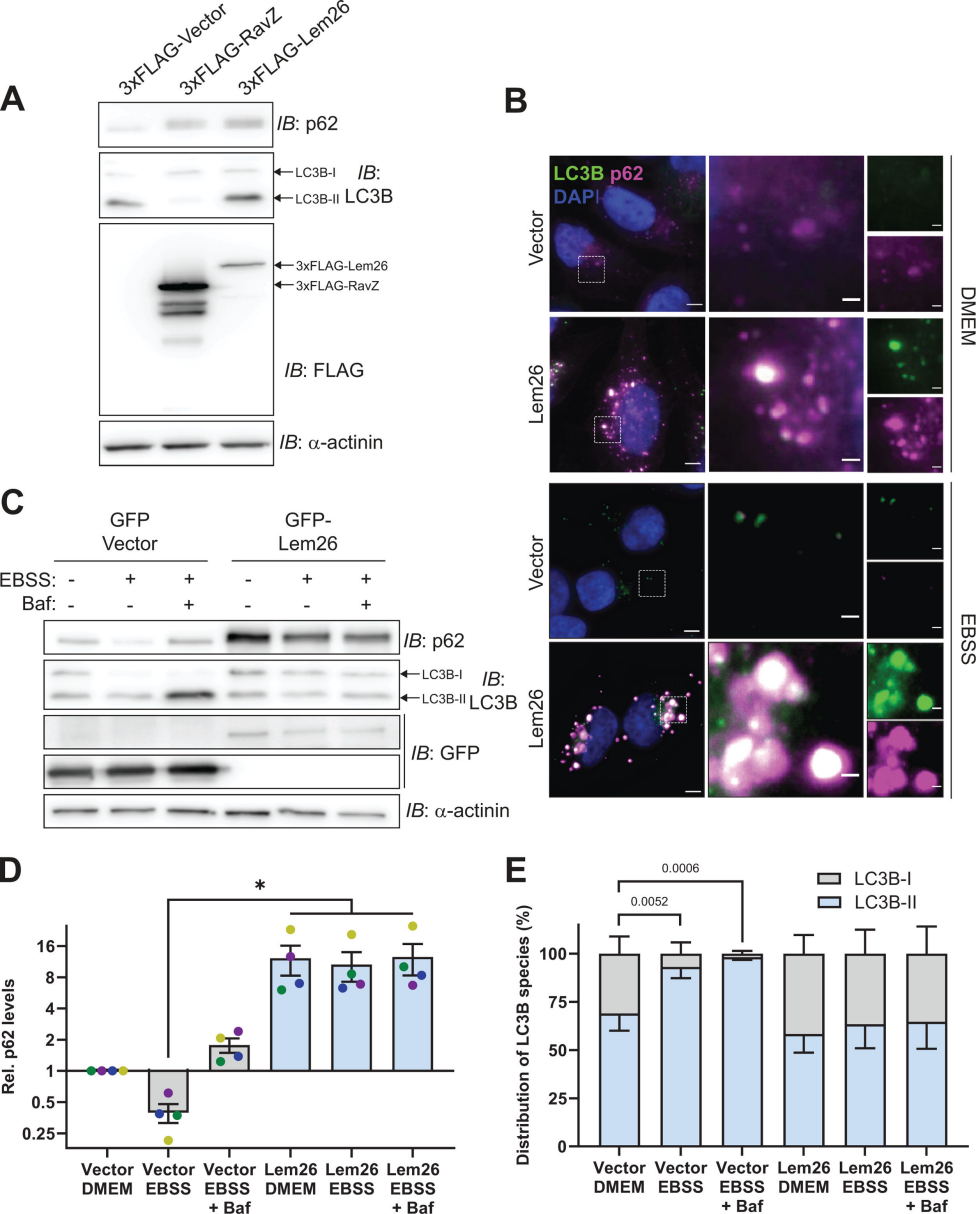
The effector Lem26 inhibits basal and starvation-induced turnover of p62 as well as starvation-induced LC3B lipidation in mammalian cells. (**A**) Immunoblot assessing levels of p62 and LC3B from HeLa cells transiently transfected with 3×FLAG-tagged RavZ or Lem26 under nutrient-rich conditions. (**B**) Immunofluorescence of p62 and LC3B from HeLa cells stably expressing free GFP or GFP-Lem26 and cultured in nutrient-rich or -depleted (EBSS) medium for 8 h. Scale bars = 5 and 1 μm for the main images and insets, respectively. (**C**) Representative immunoblots assessing levels of p62 and LC3B from HeLa cells stably expressing free GFP or GFP-Lem26 under nutrient-rich or -depleted medium ± bafilomycin for >8 h. (**D**) Quantification of p62 levels from panel **C** and replicate experiments. Data points represent p62 levels (relative to the vector + Dulbecco’s modified Eagle medium [DMEM] control, with each sample normalized to relative actin levels) from *n* = 4 independent (color-coded) experiments, with bars representing means ± SEM. Statistical significance was determined using Kruskal-Wallis with Dunn’s multiple comparisons test. *P < 0.05. (**E**) Quantification of lipid-conjugated (LC3B-II) and unconjugated (LC3B-I) species of LC3B from panel **C** and replicate experiments. Bars represent means ± SD from *n* = 4 independent experiments. Statistical significance was determined using ordinary two-way ANOVA with Dunnett’s multiple comparisons test, with each sample mean compared to the respective control samples (DMEM) for either vector or Lem26-expressing cells. Exact *P*-values are shown.

Given that Lem26 expression in yeast resulted in a level of autophagy inhibition equivalent to that of the positive control protein RavZ, we decided to further explore Lem26 function. To determine whether Lem26 can also inhibit autophagy in mammalian cells, HeLa cells transiently transfected with 3×FLAG-tagged Lem26 were examined for autophagy inhibition by measuring the levels of the autophagy adapter protein and substrate p62. The p62 protein is targeted by autophagy for degradation in lysosomes (31), so determining p62 protein levels in cells is an assay used to measure autophagic flux. HeLa cells producing either Lem26 or RavZ displayed a significant increase in p62 levels relative to the vector control, indicating defective turnover of p62 during nutrient-rich conditions (Fig. 2A). To better characterize the potential anti-autophagy activity of Lem26 during nutrient limitation, HeLa cell lines stably producing GFP-Lem26 or harboring the vector control were subjected to starvation in Earle’s Balanced Salt Solution (EBSS). Bafilomycin A1 was added to control wells to prevent lysosomal degradation of autophagic substrates. Starvation-dependent turnover of p62 was evident in control cells (Fig. 2B through D). In cells producing GFP-Lem26, there was a dramatic increase in p62 levels that did not decrease when autophagy was induced by nutrient limitation in EBSS (Fig. 2C and D), which indicates Lem26 expression inhibited autophagy. In addition, autophagy was assessed by measuring lipidation of LC3B, which is a mammalian homolog of the yeast ATG8 protein (10). Compared to control cells, starvation-dependent accumulation of the lipidated LC3B-II protein was not observed in HeLa cells producing GFP-Lem26 upon nutrient deprivation and bafilomycin A1 treatment (Fig. 2C and E). These data collectively indicate that Lem26 production is sufficient to inhibit autophagy in mammalian cells.

### Lem26 is not essential to prevent autophagy-mediated inhibition of *Legionella* replication

In addition to Lem26, it has been shown that the effector protein RavZ (8) and the SidE family of effectors (32) can interfere with autophagic recognition of the vacuole containing *Legionella*. Thus, we created a triple mutant of *Legionella* (JV6113 ∆*ravZ*, ∆*lem26*) where all the genes encoding the SidE family of effectors had been deleted in addition to the genes *ravZ* and *lem26*. We compared intracellular replication of this triple mutant in THP1 human macrophage-like cells to the parental strain called JV6113 that has genes encoding all the SidE family of effectors deleted (32) and the double mutant (JV6114 ∆*ravZ*) (Fig. S1). These data demonstrate that there is no difference in intracellular replication of the triple mutant having *lem26* deleted compared to either the parental strain JV6113, the JV6113 ∆*ravZ* strain, or the WT control strain Lp02. By contrast, the Dot-/Icm-deficient strain Lp03, having a loss-of-function mutation in the *dotA* gene, displayed a severe intracellular replication defect. Thus, *lem26* is not essential for preventing autophagy-mediated inhibition of *Legionella* replication, which indicates additional effectors that prevent autophagic recognition of the vacuole are likely still produced by this triple mutant.

### Lem26 inhibits an early stage of the autophagy pathway

**Fig 3:**
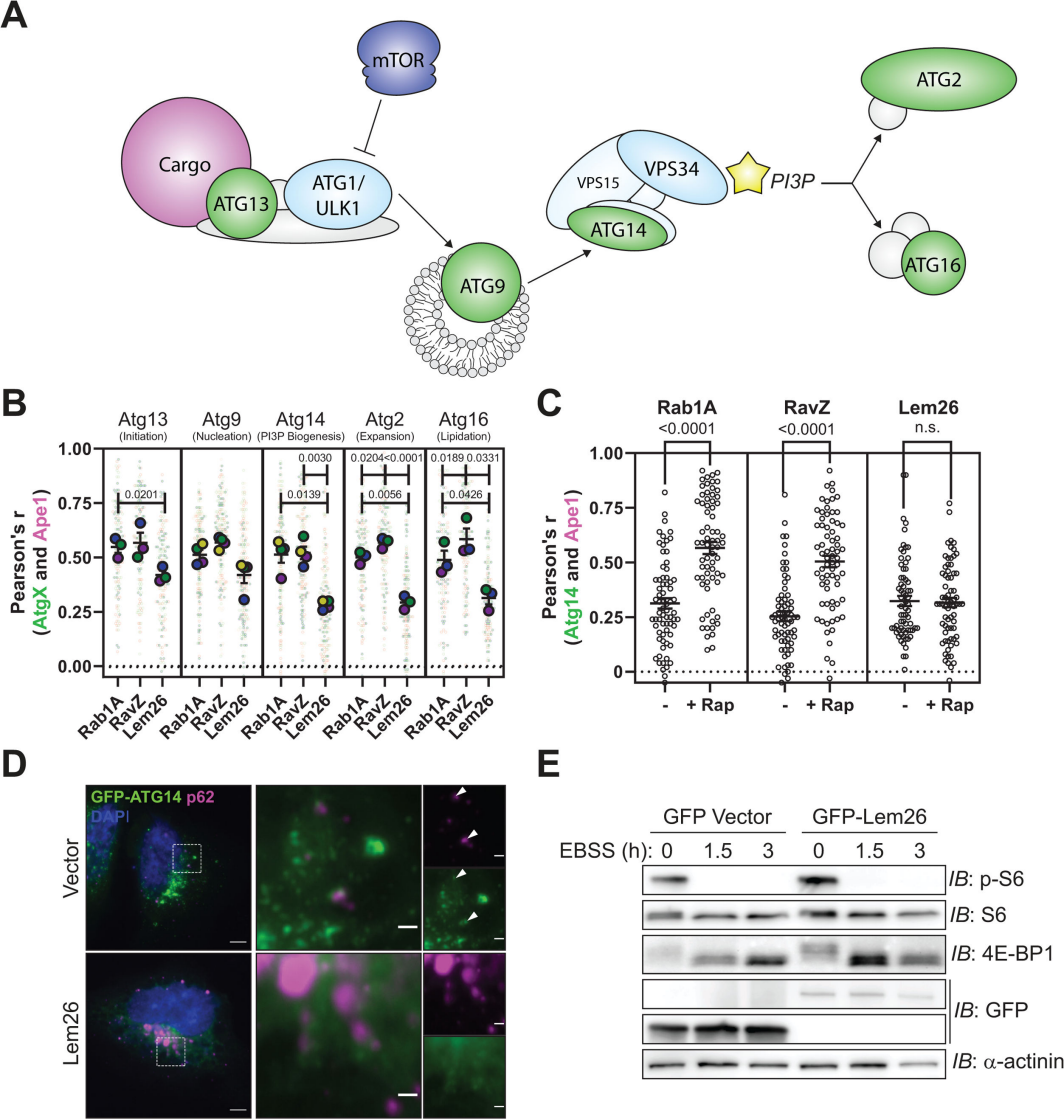
Hierarchical analysis of ATG protein recruitment demonstrates that Lem26 prevents the recruitment of ATG14 and downstream proteins to autophagic targets in yeast and mammalian cells. (**A**) Schematic of the hierarchical recruitment of autophagy-related proteins. (**B**) Assessment of autophagy protein recruitment in *S. cerevisiae* expressing Rab1A, RavZ, or Lem26 after 4 h of rapamycin exposure. SuperPlot data points (darker, large circles) represent the average colocalization (*n* > 50 cells per independent experimental sample) between each autophagy protein and Ape1 from *n* = 3–4 independent experiments, with bars representing means ± SEM. Statistical significance was determined using one-way ANOVA with Geisser-Greenhouse correction and Tukey’s multiple comparisons test. Exact *P*-values are shown. (**C**) Assessment of Atg14 recruitment in *S. cerevisiae* expressing Rab1A, RavZ, or Lem26 before and after 4 h of rapamycin exposure. Individual data points represent the colocalization between Atg14 and Ape1 from individual cells within one independent experiment (*n* = 69 cells/condition), with bars representing means ± SEM. Statistical significance was determined using one-way ANOVA Kruskal-Wallis test and Dunn’s multiple comparisons test. (**D**) Immunofluorescence of p62 and plasmid-expressed GFP-ATG14 from HeLa cells stably expressing 3×FLAG-APEX2 vector or 3×FLAG-APEX2-Lem26. Scale bars = 5 and 1 μm for the main image and insets (dashed box), respectively. Arrowheads denote colocalized p62 and GFP-ATG14. (**E**) Representative immunoblot of S6 and 4E-BP1 from lysates of HeLa cells stably expressing GFP-Lem26 or vector control during nutrient-rich (0 h) and nutrient-depleted (1.5 and 3 h in EBSS) conditions.

Autophagy-related proteins have spatial and temporal roles in the autophagy pathway, which leads to a semi-hierarchical recruitment of these proteins to autophagic substrates (Fig. 3A) (33, 34). Thus, to better understand Lem26 inhibition of autophagy, the recruitment of fluorescently tagged ATG proteins to an autophagic target was examined in yeast. These data showed a defect in the recruitment of GFP-ATG14, GFP-ATG2, and GFP-ATG16 to the autophagic substrate protein Ape1 in yeast producing Lem26 (Fig. 3B and C). By contrast, production of the effector RavZ, which disrupts autophagy at a late stage, did not affect localization of these ATG proteins. Similarly, production of Lem26 in mammalian cells disrupted recruitment of GFP-ATG14 to puncta containing p62 (Fig. 3D). Lem26 production in mammalian cells did not influence the kinase activity of proteins in the mTOR pathway, which regulates autophagy, as determined by measuring the phosphorylation of the mTOR substrates 4E-BP1 and S6 (Fig. 3E) and visualization of TFEB localization (Fig. S2B) (35). Lastly, Lem26 production in mammalian cells did not disrupt secretion of an alkaline phosphatase reporter protein (Fig. S3A) (36), cathepsin D maturation (Fig. S3B and C) (37), or LC3B lipidation and TFEB nuclear localization upon exposure to a TRPML1 agonist (Fig. S2A and B) (38), which indicates that membrane transport through the canonical secretory and endocytic pathways as well as the non-canonical conjugation of ATG8 onto single-membranes (CASM) pathways are not affected by Lem26. Thus, Lem26 has an activity that specifically disrupts canonical autophagy at an early stage in this pathway.

### A functional ADP-ribosyltransferase domain in Lem26 is essential for autophagy inhibition

**Fig 4:**
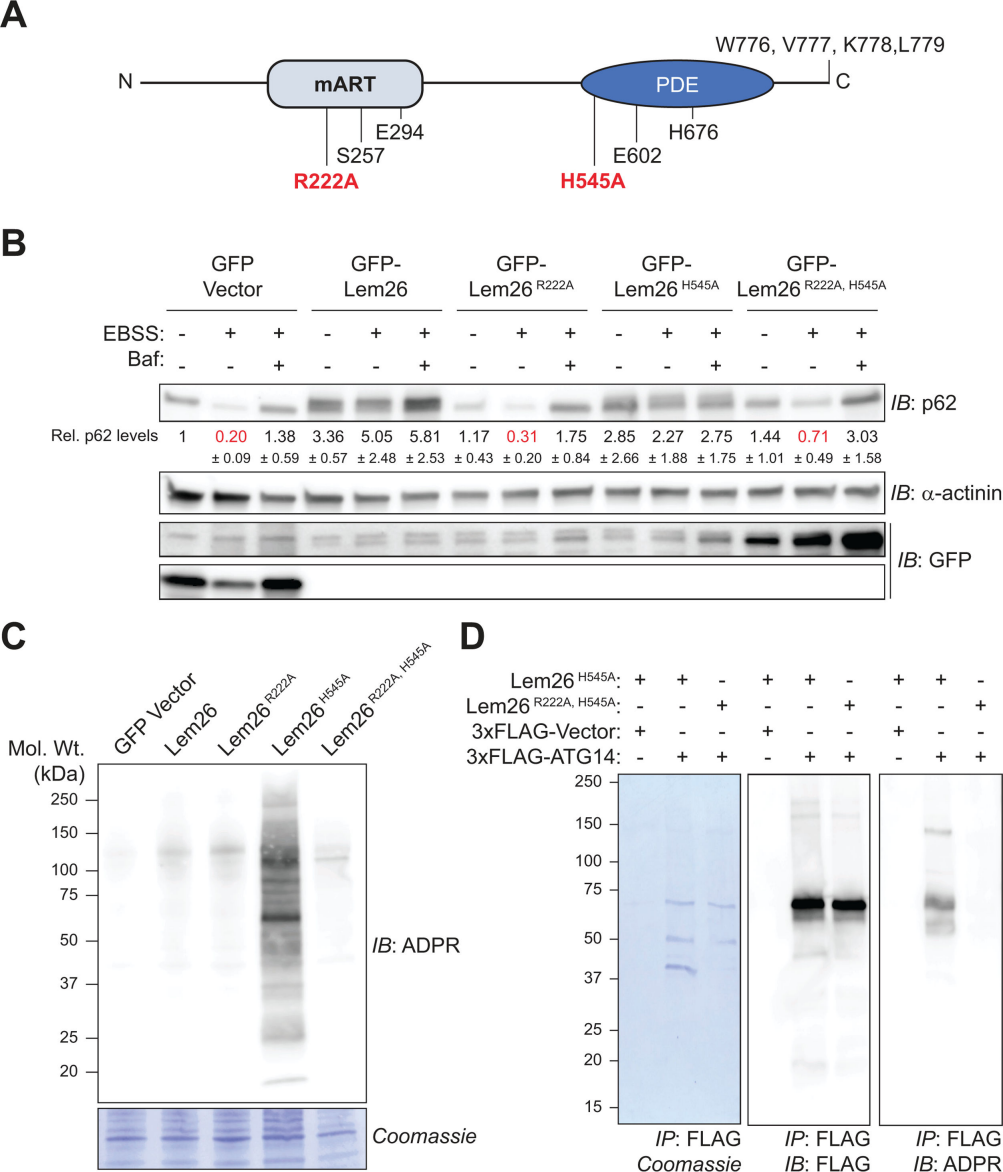
Lem26 possesses an mART domain required for autophagy inhibition and modification of autophagy proteins. (**A**) AlphaFold-predicted organization of the Lem26 mART and PDE domains and the presence of a WxxL motif at the C-terminus. Mutated residues are shown in red. (**B**) Representative immunoblot and quantification of p62 levels from HeLa cells stably expressing the GFP vector or GFP-tagged Lem26 mutants. Numbers denote p62 levels ± SD (relative to the vector + DMEM control, with each sample normalized to relative actin levels) from *n* = 3 independent experiments. (**C**) Representative immunoblot of ADP-ribosylated proteins, detected using an ADP-ribose (ADPR)-binding reagent (MABE 1016) from lysates obtained from HeLa cells stably expressing the GFP vector or each GFP-tagged Lem26 variant. (**D**) Representative Coomassie staining and immunoblots of plasmid-transfected and immunoprecipitated 3×FLAG-ATG14 from HeLa cells stably expressing Lem26^H545A^ or the catalytically dead Lem26^R222A, H545A^ variant.

A predicted Lem26 (UniProt Q5ZSJ4) structure was generated using AlphaFold (39, 40). Two distinct enzymatic domains were identified with high confidence, which were an N-terminal mono-ADP-ribosyltransferase (mART) domain and a C-terminal phosphodiesterase (PDE) domain (Fig. 4A). Mutants of Lem26 having conserved active-site residues changed to alanine were generated to determine if these enzymatic domains were important for autophagy inhibition. The Lem26^R222A^ mutant should be defective in ART activity, the Lem26^H545A^ mutant should be defective in PDE activity, and the Lem26^R222A, H545A^ mutant should lack both enzymatic activities. The p62 turnover assay was used to assess autophagy inhibition by these Lem26 mutants. Normal p62 turnover was observed in control cells and cells producing Lem26^R222A^ or Lem26^R222A, H545A^, whereas p62 accumulation was observed in cells producing WT Lem26 or the Lem26^H545A^ mutant (Fig. 4B). Thus, a functional mART domain was necessary for autophagy inhibition by Lem26, and the PDE domain was not essential, which suggests that Lem26 modifies a host protein important for autophagy using an ART activity.

### Host autophagy proteins are targets for Lem26-mediated posttranslational modification

Given the importance of the mART domain in Lem26 for autophagy inhibition, potential host targets were identified from cells producing Lem26 using an ADPR affinity reagent, which consists of an engineered Af1521 ADPR-binding protein fused to a rabbit Fc tag so that the affinity reagent can be used for immunoassays. This ADPR affinity reagent identified host proteins in lysates of mammalian cells producing the PDE-deficient Lem26^H545A^ protein (Fig. 4C). The absence of an ADPR signal in lysates producing the WT Lem26 protein suggests that the PDE domain further modifies ADP-ribosylated substrates after post-translational modification by the mART domain, which would be consistent with the PDE domain cleaving the phosphodiester bond in the ADPR moiety (41). Host proteins isolated from cells producing Lem26^H545A^ using the ADPR affinity reagent were identified by mass spectrometry. The top hits from this analysis included p62 (SQSTM1), VPS15 (PIK3R4), VPS34 (PIK3C3), and ATG16L1, which are proteins that regulate autophagy—two of which belong to the PI3KC1 complex that includes ATG14 (Fig. S4A). To determine if Lem26 can modify proteins in the autophagy pathway, 3×FLAG-tagged versions of ATG14 and VPS34 were produced in cells with different Lem26 proteins. ADP-ribosylation of ATG14 and VPS34 was detected in cells producing Lem26^H545A^ (Fig. 4D; Fig. S4B). Thus, the Lem26 protein has ART activity that can post-translationally modify autophagy proteins *in vivo*.

### Lem26 activity is activated upon interaction with membranes containing lipid-conjugated ATG8 proteins

**Fig 5:**
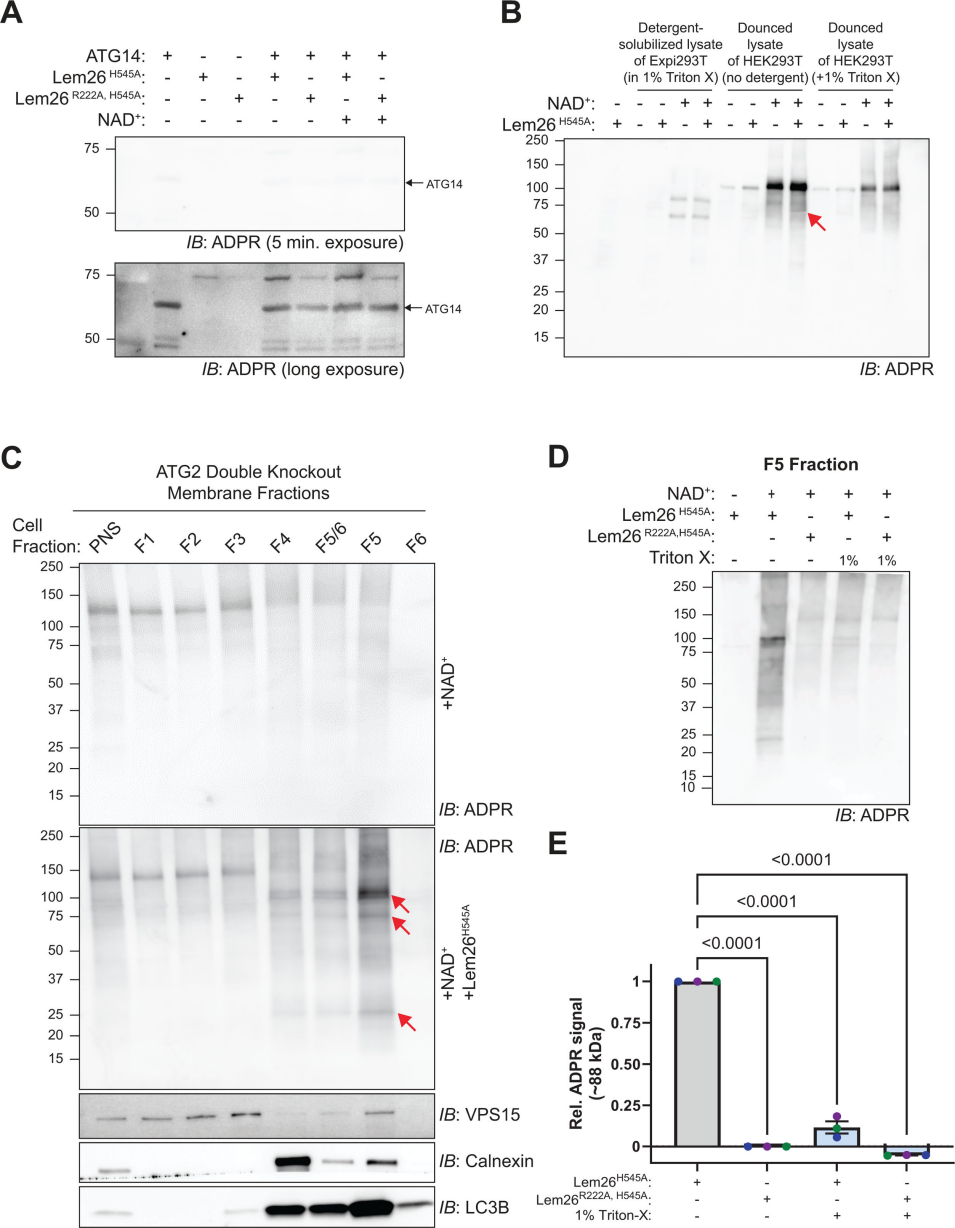
A detergent-sensitive, host component is required for the *in vitro* ADP-ribosylation activity of Lem26. (**A**) Normal and long-exposure immunoblots of the ADPR signal from 12 h *in vitro* reactions with 100 nM Lem26, 1 mM NAD+. Samples were loaded in a 7.5% SDS-PAGE gel. (**B**) Immunoblot of the ADPR signal from 4 h reactions with 100 nM Lem26, 1 mM NAD+, and HEK/Expi 293T cell lysates prepared in 1% Triton-X or mechanically disrupted via Dounce homogenization followed by addition of buffer or a final concentration of 1% Triton-X. The red arrow indicates a Lem26-dependent ADPR signal. (**C**) Representative immunoblot of membrane fractions from ATG2 DKO HEK293T cells grown under nutrient-rich conditions. Each fraction received either NAD+ alone to assess the background activity from endogenous ARTs or both NAD+ and Lem26_H545A_. After 4 h, reaction samples were probed for ADPR signal and for the presence of VPS15, calnexin, and LC3B. The red arrows indicate a Lem26-dependent ADPR signal. (**D**) Immunoblot of the ADPR signal from 4 h reactions with 100 nM Lem26, 1 mM NAD+, and the F5 membrane fraction in Fig. 3A with and without addition of 1% Triton-X. (**E**) Quantification of relative ADPR signals near the 88 kDa from each sample from *n* = 3 independent experiments utilizing the F5 fraction from two independent membrane fractionations. Statistical significance was determined using ordinary one-way ANOVA with Dunnett’s multiple comparisons test.

Biochemical assays were used to determine if Lem26 directly modifies host proteins using the mART domain. To detect ART activity, we utilized the ADPR affinity reagent (same as in Fig. 4C and D) in an immunoblot assay to determine if an ADP ribosyl group had been added to a purified target protein after incubation with Lem26 and NADH. No ART activity was detected when purified Lem26^H545A^ and ATG14 were incubated together with NADH *in vitro* (Fig. 5A). The lack of *in vitro* activity suggested that Lem26 ART activity required a cellular co-factor. The addition of a detergent-soluble extract from mammalian cells did not stimulate Lem26^H545A^ ART activity; however, ART activity was detectable (red arrow) when extracts from mechanically disrupted cells were added to the reaction (Fig. 5B). Subsequent fractionation of membranes from mechanically disrupted cells (13, 42) revealed that membranes enriched for ER and autophagosomal proteins activated Lem26^H545A^ ART activity (red arrows) *in vitro* (Fig. 5C). Importantly, high levels of Lem26^H545A^ ART-stimulating activity correlated with the amount of LC3B-II, which is the lipidated form of LC3B conjugated to the membranes. Detergent solubilization of these membranes abolished Lem26^H545A^ ART activity (Fig. 5D and E). These data suggest that Lem26^H545A^ ART activity is activated upon binding to autophagy-associated membranes.

**Fig 6:**
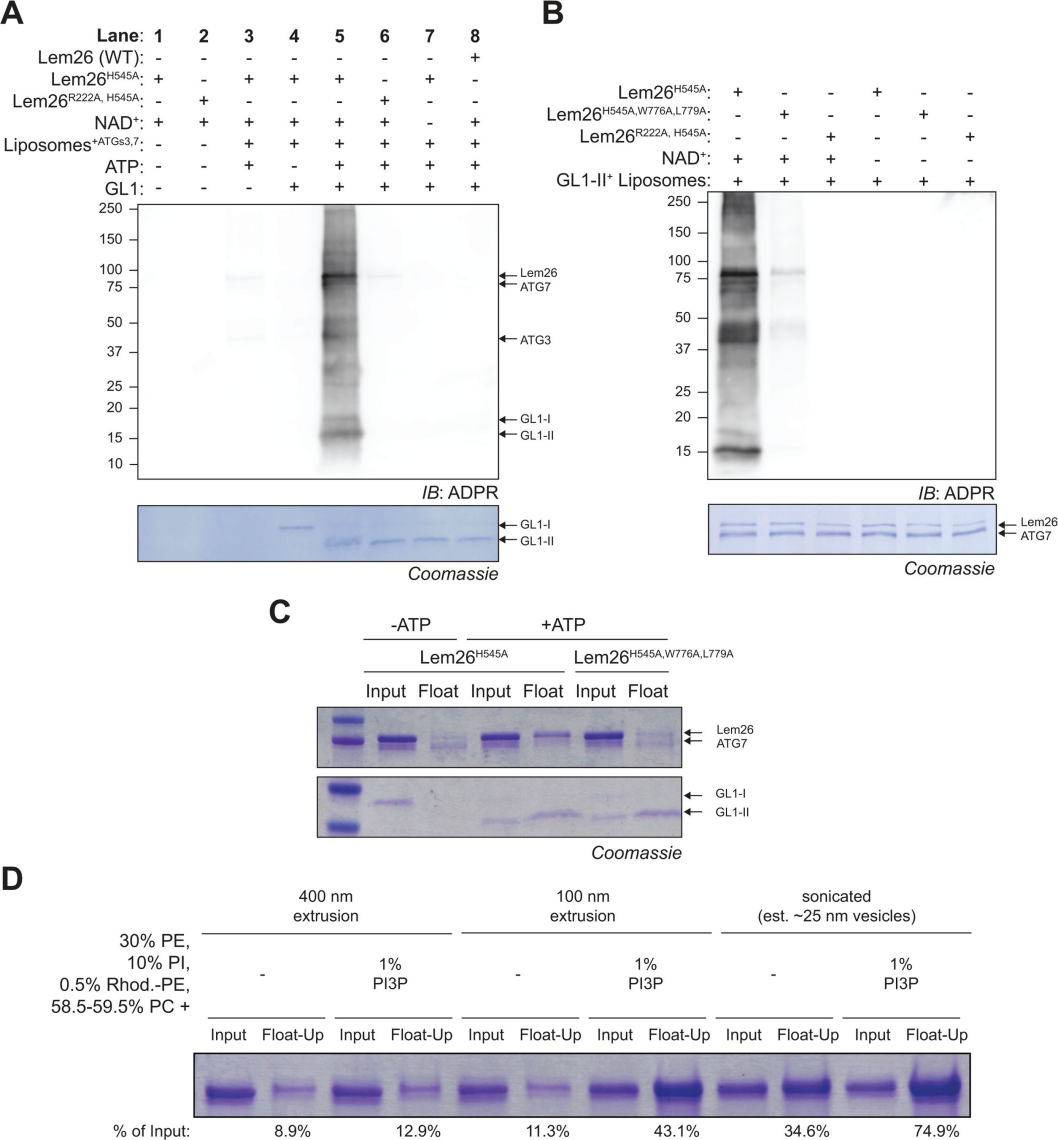
The enzymatic activity of Lem26 is triggered by membrane recruitment via interaction with ATG8, membrane curvature, and PI3P. (**A**) Representative immunoblot of ADPR signals from 4 h *in vitro* reactions performed after 1 h of GL1 lipidation. Complete reactions (Lane 5) contained 3 mM of liposomes with 15 µM GL1, 1 µM ATG7, 1 µM ATG3, 0.5 mM DTT, 0.5 mM ATP, 100 nM Lem26, and 1 mM NAD. Individual components were omitted and replaced with buffer during the lipidation or ART reactions in other lanes. Each reaction was subjected to Western blot and visualized for ADPR signals followed by Coomassie staining of the same membrane to visualize GL1 lipidation. (**B**) Representative immunoblot of ADPR signals from 4 h *in vitro* reactions performed on the same pool of liposomes subjected to 1 h of GL1 lipidation, as described in panel **A**. Each reaction was subjected to Western blot and visualized for ADPR signals followed by Coomassie staining of the same membrane to verify identical levels of Lem26 mutants. (**C**) Coomassie stain of a membrane flotation assay showing input and float fractions of Lem26 mutants incubated with complete (+ATP) and incomplete (−ATP) lipidation reactions. Samples were subjected to 4 h gradient centrifugation at 48,000 rpm in a Beckmann SW55 rotor to separate membrane-bound (Float) and unbound proteins. (**D**) Coomassie stain of a membrane flotation assay showing input and float fractions of Lem26 incubated with liposomes extruded through different pore-sized membranes ± 1% PI3P. No other proteins were included in these samples. Samples were subjected to gradient centrifugation, as described in panel **C**.

An *in vitro* liposome-based assay was used to directly test whether Lem26 ART activity was stimulated by autophagic membranes. The purified mammalian ATG8 homolog GABARAPL1 (GL1) was directly conjugated to liposome membranes *in vitro* upon the addition of the conjugating enzymes ATG3 and ATG7 and ATP to generate autophagosome-like membranes (hereafter referred to as synthetic autophagosomes). The addition of Lem26^H545A^ and NADH to these synthetic autophagosomes resulted in ADP-ribosylation of the autophagosome-associated proteins *in vitro* (Fig. 6A; Fig. S5B). Importantly, conjugation of GL1 to the membranes was essential to stimulate Lem26^H545A^ ART activity as omission of ATP, but inclusion of all other components failed to stimulate ADP-ribosylation of substrates. Additionally, a membrane flotation assay showed Lem26 binding to these synthetic autophagosomes by a process that required GL1 conjugation to membranes (Fig. S5A). Thus, Lem26 ART activity is activated upon binding to autophagic membranes.

### Lem26 contains an ATG8-interaction motif important for membrane recruitment

The flotation assay (Fig. S5A) suggested Lem26 has the capacity to bind directly to autophagic membranes. Bioinformatic analysis identified a canonical ATG8-interaction motif (AIM) consisting of (W/F/Y)xx(L/I/V) where the first (aromatic) and fourth (branched, hydrophobic) residues in the motif mediate binding to a conserved hydrophobic pocket in the ATG8 proteins (10). Additionally, the program OmegaFold was used to identify potential regions in Lem26 that could interact with ATG8 proteins. This *in silico*-based approach predicted that the C-terminal residues in Lem26 (W776, V777, K778, and L779) resemble a motif found in AIMs that binds to a conserved hydrophobic pocket of yeast ATG8, human LC3B, and human GL1 (Fig. S6). A Lem26 mutant was generated where three predicted critical C-terminal residues were changed to alanine (Lem26^H545A, W776A, and L779A^). Flotation assays showed that the Lem26^H545A, W776A, and L779A^ mutant was defective for binding to synthetic autophagosomes (Fig. 6C) and that ATG8-dependent activation of ART activity was significantly reduced (Fig. 6B). Thus, Lem26 has a C-terminal AIM important for regulation of protein function.

We also generated liposomes with high membrane curvature that contained PI3P to mimic pre-autophagosomal membranes. These liposomes were used to determine if Lem26 activation was enhanced by either PI3P or membrane curvature, which contribute to the binding of other autophagy-related proteins and effectors (28, 43, 44). Purified Lem26 was detected on liposomes containing PI3P or high membrane curvature on a flotation gradient (Fig. 6D), which suggests that Lem26 also has properties that can detect biophysical properties of pre-autophagosomal membranes. Lem26 ART activity was detected *in vitro* upon incubation with vesicles with high membrane curvature containing PI3P but no conjugated ATG8; however, the level of activation was less than that detected using vesicles that contained conjugated ATG8 protein or both ATG8 and PI3P (Fig. S7). In summary, these data suggest that Lem26 targeting of host substrates is tightly regulated and is stimulated by the unique properties of early autophagosomal membranes that include the presence of lipid-conjugated ATG8 proteins, high levels of PI3P, and high membrane curvature.

## DISCUSSION

Bacterial pathogens that have evolved to survive within eukaryotic cells pose a unique source of knowledge for how host processes are regulated. Several of the Dot/Icm effectors translocated into host cells by *Legionella pneumophila* post-translationally modify conserved host proteins to manipulate membrane transport pathways and promote replication within the LCV (2, 45). By expressing individual effectors in yeast and using both a visual assessment and immunoblot-based quantification of GFP-ATG8 vacuolar traffic and processing, we identified several *Legionella* effector proteins that potentially play a role in inhibition of host autophagy.

Clearly, there are multiple proteins encoded by *Legionella* that can interfere with autophagy. In addition to the previously identified effectors such as RavZ that directly target core autophagy proteins, several *Legionella* effectors that modulate host membrane transport by modifying host determinants could indirectly influence the autophagy pathway. For example, Lpg0696 (AnkX) is a phosphocholine transferase that targets Rab1 and several other Rab proteins, causing defects in the secretory pathway, which plays a role in autophagosome biogenesis (46, 47). Lpg1426 (VpdC) is a phospholipase that generates lysophospholipids within the vacuolar membrane (48). Given the importance of the secretory pathway and phospholipid synthesis (49) to autophagosome biogenesis, Lpg0695 (AnkX) and Lpg1426 (VpdC) may contribute to the modulation of autophagy by *Legionella*. Despite the large number of effectors identified previously, there has not been a systematic screen for *Legionella* effectors that specifically inhibit autophagy, which was the impetus for conducting our screen in yeast.

In addition to Lem26, this yeast screen identified Lpg1137 as a potential inhibitor of autophagy. Production of Lpg1137 reduced the total percentage of cells that had increased GFP fluorescence in the vacuole (Fig. 1B). Intriguingly, this effector was previously characterized as a serine protease that can cleave Syntaxin-17 (23), which is a mammalian protein involved in autophagosome-lysosome fusion, but is absent in yeast (50). Nonetheless, Lpg1137 did not produce a significant effect on the cleavage of GFP-ATG8 (Fig. 1D). It may be possible that this effector, as well as Lpg0195, which was a hit in our yeast screen that did not affect proteolytic cleavage of GFP-ATG8, may target other eukaryotic proteins and indirectly affect vacuolar GFP fluorescence by an unknown mechanism.

Lem26 (also known as Lpg2523) emerged as the top candidate in our screen for anti-autophagy effectors. Assays for monitoring autophagy showed that Lem26 production inhibited autophagy in both yeast (Fig. 1) and mammalian cells (Fig. 2). Furthermore, the expression of Lem26 in both yeast and mammalian cells prevented the recruitment of Atg14 to autophagic targets (Fig. 3). These data indicated that Lem26 has an activity that interferes with host autophagy.

The AlphaFold-generated structure for Lem26 aligns with proteins that contain either an mART or PDE domain. Mutagenesis of a predicted catalytic residue in the mART domain (Lem26^R222A^) resulted in the loss of autophagy-inhibiting activity, but mutagenesis of a predicted catalytic residue in the PDE domain did not (Fig. 4B). Although this raises the question of the importance and function of the PDE domain, this is not entirely unprecedented as members of the SidE effector family, which also possess both mART and PDE domains, retained the ability to interfere with the interaction between ubiquitin and the autophagy receptor p62 when the catalytic histidine in their PDE domains was mutated (25). It is possible that the PDE domain in Lem26 acts sequentially with the ART domain, and after host targets are ADP-ribosylated, the PDE domain could cleave the phosphodiester bond in the ADPR moiety to leave a phosphoribosyl modification. By contrast, the role of the PDE domain in SidE is to mediate the non-canonical, phosphoribosyl-linkage between the ADP-ribosylated substrate protein ubiquitin and host targets such as reticulon-4 and Rab33 through the addition of phosphoribosyl-ubiquitin (32, 51).

HHpred analysis (52) of the Lem26 PDE domain reveals a high degree of structural similarity with the PDE domains of SidE, SdeA, SdeC, and SdeD, each with E-values less than 1e-37. Although there is a high degree of similarity between the PDE domains of Lem26 and the SidE effector family, previous data showed that the PDE domain of Lem26 was unable to hydrolyze ADPR-Ub generated by the mART domain of the SidE effector family (41). This may be due to the differences in the mART domains of Lem26 and the SidE effector family as the Lem26 mART is most similar to *C. botulinum* C2 toxin, albeit with an E-value of 0.33. Alternatively, it is possible the PDE activity in Lem26 is also tightly regulated and requires binding of Lem26 to autophagosomal membranes.

Other differences between Lem26 and the SidE family proteins include their targets as well as the mechanisms required for enzymatic activity. The SidE proteins will cause ADP-ribosylation of ubiquitin in solution, which indicates that substrate binding regulates activity. By contrast, our data show that Lem26 ART activity requires effector binding to autophagosomal membranes. Membrane recruitment is mediated by an AIM domain in Lem26 that confers interactions with lipid-conjugated ATG8 proteins. Additionally, the lipid PI3P and membrane curvature enhance Lem26 ART activity. These biochemical signatures define the early membranes that generate autophagosomes (11) and explain the ability of Lem26 to specifically inhibit the autophagy pathway and not other pathways (CASM, secretory, and endocytic) (Fig. S2 and S3). Thus, Lem26 shares some features with RavZ, which specifically inhibits autophagy by being activated upon binding to autophagosomes containing conjugated ATG8 proteins (8, 28). Further studies are required to refine our understanding of interfacial activation of Lem26 upon binding to autophagosomal membranes.

Our *in vitro* data demonstrate that membranes containing lipidated ATG8 proteins are critical for activation of Lem26; however, the *in vivo* Lem26 autophagy inhibition data indicate that autophagy disruption occurs before canonical ATG8 conjugation (Fig. 4B). These data suggest that small amounts of lipidated ATG8 may be sufficient to promote activation of Lem26, and this leads to disruption of upstream autophagy events. Previous studies have shown the localization of ATG8 in the absence of ATG1, ATG13, and ATG7 proteins (33), which suggests an alternative pathway for initiating ATG8 conjugation. This early ATG8-lipidation event may amplify the recruitment of autophagy proteins to pre-autophagosomal membranes. Consistent with this hypothesis, it has been shown that the upstream autophagy proteins ATG1/ULK1 and ATG14 contain ATG8-/LC3-interacting motifs/regions (AIMs/LIRs), and point mutations within these AIMs/LIRs result in defects in both their own recruitment as well as in autophagic flux (53, 54). Thus, it is thought that early ATG8 lipidation on a seed membrane assists in the recruitment of the ATG1/ULK1 and PI3KC1 complexes required for the canonical assembly of the autophagy machinery. It is likely that Lem26 has evolved a similar mechanism to recognize early ATG8-lipidation events to modify host autophagy proteins needed to generate functional pre-autophagosomal structures.

From these data, we propose a model whereby Lem26 is recruited to early autophagosomal membranes by sensing conjugated ATG8 in conjunction with membrane curvature and PI3P. The ART activity of Lem26 is activated at this stage, and upon activation, Lem26 modifies multiple proteins associated with these early autophagosomal structures, which includes proteins essential for the biogenesis of a mature autophagosome. Thus, Lem26 stalls autophagosome biogenesis at an early stage. Understanding how Lem26 functions during infection and how Lem26 function complements the activities of other *Legionella* effectors that block autophagy should reveal the complex and functionally redundant mechanisms that enable this intracellular pathogen to avoid autophagic recognition in evolutionarily diverse eukaryotic hosts.

## MATERIALS AND METHODS

### Cell lines, yeast strains, and culture conditions

HeLa cells were cultured at 37°C and 5% CO_2_ in DMEM (ThermoFisher, 11965092), supplemented with 5% FBS (Sigma, F4135). For transfection, 1 × 10^5^ cells were seeded into 12-well plates overnight and transfected with 0.5–1 μg of each plasmid using TransIT-LT1 Transfection Reagent (Mirus Bio) for 24–48 h. In the case of immunoprecipitation, 5 × 10^6^ cells were seeded into 15 cm dishes and transfected as mentioned previously, albeit with 20 μg of the plasmid. For experiments involving starvation, cells were washed 3–5× in PBS and incubated in EBSS (ThermoFisher 24010043) with or without bafilomycin A1 (Cayman Chemicals 11038), SBI-0206965 (SIGMA SML1540), or SAR405 (MedChem Express HY-12481), as indicated in the figure legends.

Yeast strains (Table 1) were constructed in W303α (MATα, ade2-1 can1-100 his3-11,15 leu2-3,112 trp1-1 ura3-1) by homologous recombination of gene-targeted, polymerase chain reaction (PCR)-generated DNAs using the method of Longtine et al. (55), via amplification of a LEU1 insertion cassette, or the method of Zhang et al. (56). Genomic modifications were confirmed by PCR amplification using external primers flanking the modified locus. Cells were grown at 30°C, shaking at 225 rpm, in either yeast extract-peptone-dextrose or in standard synthetic complete medium (MP Biomedicals, Sunrise Science Products) lacking nutrients required to maintain selection for auxotrophic markers and/or plasmids. For the induction of autophagy, cells were cultured to the early log phase and exposed to 200 μg/mL of rapamycin (MedChem Express) ±1 µM CMAC (Thermo Scientific C2110) for 4 h at 30°C.

**TABLE 1:** Yeast strains used in this study

Name	Genotype	Origin	Generation
PMCPL464	W303α (alpha), GFP-Atg8 (endogenously tagged)	Courtesy of Philip J. Mannino and C. Patrick Lusk	Integration through PCR product transformation using N-ICE plasmids (56)
KPY5	PMCPL464, atg1Δ::leu2p-LEU2	This study	Integration through PCR product transformation
W303α	MATα, ade2-1 can1-100 his3-11,15 leu2-3,112 trp1-1 ura3-1	EUROSCARF	
KPY8	W303α (alpha), Atg2-2xGFP::Ura	This study	Integration through PCR product transformation
KPY9	W303α (alpha), Atg16-2xGFP::Ura	This study	Integration through PCR product transformation
KPY11	W303α (alpha), Atg13-2xGFP::Ura	This study	Integration through PCR product transformation
KPY12	W303α (alpha), Atg14-2xGFP::Ura	This study	Integration through PCR product transformation
KPY13	W303α (alpha), Atg9-2xGFP::Ura	This study	Integration through PCR product transformation

### Construction of *Legionella* mutants and intracellular replication assays

*Legionella pneumophila* strains generated in this study were constructed in strain JV6113, which is a strain of Lp02 that had the genes encoding the SidE family of effectors deleted (32). Strains were cultured at 37°C on charcoal *N*-(2-acetamido)−2-aminoethanesulfonic acid-buffered yeast extract agar (CYE) as described previously (57), 100 µg/mL thymidine, and ± 100 µg/mL streptomycin. The *ravZ* and *lem26* genes were deleted using allelic exchange, as described previously (58, 59). In brief, deletion constructs were generated in pSR47 using 1,000 bp flanks upstream and downstream of each respective gene. Sanger-sequenced plasmids were conjugated to *L. pneumophila* via triparental mating with *E. coli* strains DH5α λpir containing the aforementioned deletion construct and *E. coli* MT607 containing the pRK600 plasmid that provides the conjugation machinery for plasmid transfer. Sucrose-resistant, kanamycin-sensitive clones were screened via PCR to verify gene deletion.

For intracellular growth assays, THP1 cells were cultured at 2.5 × 10^5^ cells/well in RPMI, 10% FBS, and 2 mM L-glutamine and were infected at MOI = 0.1 using bacteria patched onto CYE plates from a single colony and cultured for 48 h. Following inoculation, plates were centrifuged at 350 × *g* for 5 min. prior to incubation at 37°C, 5% CO_2_ for 30 min., after which, cells were washed 3× in PBS. At various time points, host cells were subjected to hypotonic lysis via the addition of 500 µL of sterile water followed by 20× vigorous pipetting followed by dilution plating and subsequent enumeration of *Legionella* colony-forming units.

### Microscopy-based screening and colocalization experiments

W303α expressing endogenously tagged GFP-Atg8 (strain PMCPL464, courtesy of Philip Mannino and C. Patrick Lusk) and plasmid-encoded effectors were subcultured in SC-Leu medium (MP Biomedicals) to early log phase and were exposed to 200 μg/mL of rapamycin and 1 µM CMAC (Thermo Scientific C2110) for 4 h. Cells were mounted in growth medium on glass slides and imaged at room temperature (RT) using a Nikon Eclipse TE2000-S inverted microscope equipped with a Spectra X light engine from Lumencor, CoolSNAP EZ 20 MHz digital monochrome camera from Photometrics and Nikon Plan Apo 100× objective lens (1.4 NA). SlideBook (version 6.2) software was used for image acquisition without deconvolution. Individual cells were scored in a binary fashion, receiving a “1” if the vacuolar GFP signal was greater than or equal to the cytosolic signal or a “0” if the cytosolic signal was greater than the vacuolar signal—as assessed by eye in an unblinded fashion.

For colocalization quantification, yeast expressing GFP-tagged autophagy proteins and plasmid-encoded RFP-Ape1 were cultured and imaged as mentioned in the prior paragraph, albeit without the addition of CMAC. Max-projection images of >50 cells each experiment and strain were assessed using the Coloc 2 plugin for the Fiji distribution of ImageJ (60) to obtain Pearson’s r. Only the averages within *n* = 3–4 independent experiments were used for statistical analyses.

### SEAP assay

HEK293T cells in 96-well plates were co-transformed with 0.05 µg pSEAP and 0.075 µg 3×FLAG-tagged constructs for 24 h. The 3×FLAG vector and the GDP-locked allele of Arf1 (Arf1_T31N_) served as negative and positive controls for secretory pathway inhibition, respectively. Cells were then washed with 1× PBS and given fresh medium for an additional 8 h after which levels of intracellular and secreted (in supernatant) alkaline phosphatase were measured using Phospha-Light SEAP Reporter Gene Assay System (Thermo Fisher T1015) per the manufacturer’s instructions. Ratios of extracellular and intracellular SEAP activity were calculated from three independent experiments.

### Immunoblotting and analysis

Cell lysates were either prepared in lysis buffer (10 mM HEPES pH 7.5, 300 mM NaCl, 0.2% Triton X-100 with EDTA-free protease inhibitor cocktail (SIGMA 11873580001) followed by the addition of 5× SDS sample buffer or were directly lysed in SDS sample buffer (2% SDS, 50 mM Tris-HCl pH 6.8, 0.1 M dithiothreitol, 0.1% bromophenol blue, and 10% glycerol). Cell lysates were boiled at 98°C for 5 min, electrophoresed through either homemade (7.5%, 10%, or 15%) or pre-cast (4%–20% Mini-PROTEAN, Bio-Rad 4568096) polyacrylamide gels, transferred onto PVDF membranes (Bio-Rad 162-0177) for 60 min at 100V, blocked with 5% BSA (Sigma A9647) or milk (American Bioanalytical AB10109-01000) for 60 min, and were subjected to immunoblotting with the following antibodies: anti-GFP (Torrey Pines Biolabs TP401, 1:5,000), anti-p62 lck ligand (BD Biosciences 610832, 1:1,000), anti-LC3B (Cell Signaling 3868S, 1:1,000), anti-actinin (BD Biosciences 612576, 1:10,000), anti-S6 Ribosomal Protein (Cell Signaling #2217, 1:1,000), anti-phospho-S6 Ribosomal Protein (Ser 235/236) (Cell Signaling #4858, 1:1,000), anti-4E-BP1 (Cell Signaling #9644, 1:1,000), and anti-pan-ADP-ribose binding reagent (Millipore Sigma MABE1016, 1:1000) for 1 h at RT or overnight at 4°C. Membranes were washed in phosphate or tris-buffered saline with 0.1% Tween 20 (PBST/TBST) before incubation with Goat anti-Rabbit or Goat anti-mouse secondary antibody (Thermo Scientific A32732 and A32728) for 1 h at RT. Finally, the membranes were washed and subsequently imaged using chemiluminescence (Cytiva RPN2209).

For densitometry measurements, anti-actinin densitometry measurements were first normalized relative to the Vector + DMEM/no treatment control. p62 densitometry measurements were then normalized to the relative anti-actinin levels across each sample. Densitometry measurements for each LC3B and cathepsin D species were normalized relative to the combined measurements of all LC3B or cathepsin D species.

### Immunofluorescence

HeLa cells were seeded onto glass coverslips in a 12-well plate at a density of 100,000 cells per well. The following day, plasmids were either transfected (Mirus TransIT-LT1) for 24 h and/or starved for 2–4 h prior to fixation in 4% PFA (Electron Microscopy Sciences 15710). Cells were permeabilized in blocking solution (PBS containing 0.05% saponin; 2% BSA) for 30 min, followed by a 1 h incubation with primary antibody: anti-ubiquitin (FK2) (Enzo Life Sciences BML-PW8810, 1:100), anti-p62 (BD Biosciences 610832, 1:300), anti-LC3 (MBL International PM036, 1:500), ATG9A (Abcam ab108338, 1:250), and anti-TFEB (Cell Signaling #37785, 1:100). Coverslips were then washed 3× with blocking solution followed by a 1 h incubation with secondary antibody (Alexa Fluor 647 A21236; Alexa Fluor 488 A11029) and DAPI at a final concentration of 2.5 μg/mL. Coverslips were washed 3× with blocking solution and were mounted and dried overnight in ProLong Glass Antifade Mountant (ThermoFisher P36984). The samples were then imaged using a Nikon Eclipse TE2000-S inverted microscope equipped with a Spectra X light engine from Lumencor, CoolSNAP EZ 20 MHz digital monochrome camera from Photometrics, and Nikon Plan Apo 100× objective lens (1.4 NA). SlideBook (version 6.2) software was used for image acquisition without deconvolution, and image analysis was performed using the Fiji distribution of ImageJ (60).

### Immunoprecipitation and proteomics analysis

HeLa cells were seeded onto 15-cm dishes, transfected with 15–20 µg of plasmid using TransIT-LT1 Transfection Reagent (Mirus Bio) for 24–48 h. Cells were washed in PBS, scraped, and lysed in 1 mL of lysis buffer (25 mM HEPES pH 7.2, 75 mM NaCl, 0.5 µM EDTA, 0.5% Triton X-100, 10% glycerol, 0.5 mM DTT, and 1 mM PMSF) with EDTA-free protease inhibitor cocktail (SIGMA 11873580001) on ice for 20 min. Samples were centrifuged at 100 × *g* for 5 min at 4°C to remove the debris. Antibody-conjugated, washed, and equilibrated beads (Dynabeads Protein G for Immunoprecipitation, Invitrogen 10003D; anti-pan-ADP-ribose binding reagent, Millipore Sigma MABE1016; Anti-FLAG M2 Magnetic Beads, Millipore Sigma M8823) were added to each sample and rocked for 2 h at RT or 4°C overnight. Beads were then washed 3× in wash buffer 1 (25 mM HEPES pH 7.2, 75 mM NaCl, 0.5 µM EDTA, 0.5% Triton X-100, 5% glycerol, 0.5 mM DTT, and 1 mM PMSF) and 2× in wash buffer 2 (25 mM HEPES pH 7.2, 75 mM NaCl, 0.5 µM EDTA, 5% glycerol, and 1 mM PMSF), with 5 min incubation for each wash step. Lastly, samples were either resuspended in SDS-PAGE sample buffer or competitively eluted with 3×FLAG peptide (Sigma F4799) per the manufacturer’s instructions. For samples submitted to the Yale W.M. Keck Foundation Proteomics Center, immunoprecipitated proteins were separated by SDS-PAGE on a 4%–20% Mini-PROTEAN TGX Precast Protein Gel (BIO-RAD 4561094), excised, and submitted for trypsin digest and protein identification.

### Protein purification

*L. pneumophila* Lem26, mouse ATG3, and human GABARAP L1 (GL1, in a truncated “pro-form” with a reactive COOH-terminal glycine to circumvent the ATG4-mediated pre-processing step in cells) were cloned into pGEX-6p and transformed into *E. coli* BL21-DE3. Transformants were sub-cultured in Luria Bertani broth to an OD of ~0.6–0.7 before induction of recombinant protein expression with 400 μM isopropyl β-D-thiogalactopyranoside (IPTG, GoldBio I2481C5) for 3–5 h. Cells were lysed using a cell disruptor in either prescission protease buffer (50 mM Tris HCl, pH 7.4, 150 mM NaCl, 1 mM EDTA, and 1 mM DTT) for Lem26 and ATG3 or thrombin buffer (20 mM Tris pH 7.5, 100 mM NaCl, 5 mM MgCl_2_, 2 mM CaCl_2_, and 1 mM DTT) for GL1 in the presence of protease inhibitor cocktail. Lysates were then cleared for debris, and the remaining supernatant was incubated with glutathione beads (SIGMA G4510) for 2–3 h at 4°C. Beads were then washed 2–3× and incubated with buffer and 25 µL prescission protease (Sigma GE27084301) or 10 µL thrombin (Sigma T6884) overnight at 4°C to remove the GST tags. Purified proteins were stored in 15% glycerol at −80°C.

### Liposome preparation and lipidation of Atg8 family of proteins

All lipids were purchased from Avanti Polar Lipids, Inc., dissolved in chloroform into 1–10 mg/mL stocks, and stored at −20°C. A final concentration of 12 mM of phospholipids (final volume of 500 uL after resuspension) containing molar percentages of 30% 1,2-dioleoyl-sn-glycero-3-phosphoethanol-amine (DOPE), 10% L-phosphatidylinositol from bovine liver (PI), 0.5% 1,2-dioleoyl-sn-glycero-3-phosphoethanolamine-N-(lissamine rhodamine B ph) (DOPE-Rhod), 58.5%–59.5% (1-palmitoyl-2-oleoyl-sn-glycero-3-phosphocholine (POPC, ± 0%–1% 1,2-dioleoyl-sn-glycero-3-phospho-(1′-myo-inositol-3′-phosphate) (18:1 PI(3)P) or L-α-phosphatidylinositol-4,5-bisphosphate (brain, porcine) (PI(4,5)P_2_) were mixed and dried to a thin film under nitrogen gas. Residual solvents were removed under vacuum for >1 h. The thin film of mixed lipids was then resuspended in 500 μL ADP-ribosylation buffer (50 mM Tris HCl, pH 7.4, 150 mM NaCl, 1 mM MgCl_2_, and 1 mM CaCl_2_) and was subjected to 7 freeze-thaw cycles. Resuspended lipids were then extruded through two polycarbonate, 200 nm membranes (Whatman) using the LipSoFast-Basic extruder (Avestin) at RT and stored on ice at 4°C. Liposomes were then sonicated with the Virsonic 600 (VirTis) microtip sonicator immediately prior to the lipidation reaction. For the *in vitro* lipidation of GL1, purified GL1 (final concentration of 15 μM), ATG3 (2 μM), ATG7 (2 μM), and sonicated liposomes (6 μM) were mixed with 1 μM DTT ± 1 μM ATP at 37°C for 60–120 min.

### *In vitro* ADP-ribosylation

Purified Lem26 (WT), Lem26^H545A^, or Lem26^R222A, H545A^ (100–500 nM) were incubated with final concentrations of 1 mM NAD (SIGMA 43410) or 100 µM biotin-NAD (TOCRIS 65-731-31U), 3 mM liposomes with lipidated Atg8 proteins, and ± 100–500 nM ATG14 (MedChem Express) in ADP-ribosylation buffer (50 mM Tris HCl, pH 7.4, 150 mM NaCl, 1 mM MgCl_2_, and 1 mM CaCl_2_) for 2–4 h at 37°C or with an additional 12 h at 16°C (for reactions using biotin-NAD). Reactions were stopped via the addition of SDS Sample Buffer (2% SDS, 50 mM Tris-HCl pH 6.8, 0.1 M DTT, 0.1% bromophenol blue, and 10% glycerol) and boiling for 10 min.

### Membrane fractionation

Membrane fractionation was performed as previously described (13). In brief, ATG2 double knockout cells from 20 15-cm dishes at 80%–90% confluency were scraped, resuspended in 1 mL sucrose homogenization buffer (50 mM Tris pH 7.4, 150 mM NaCl, 10% sucrose, and 1× EDTA-free protease inhibitor cocktail [SIGMA 11873580001]), and lysed with a 2 mL Dounce homogenizer (Sigma-Aldrich DWK8853000001) on ice. The lysates were subsequently centrifuged at 2,000 × *g* at 4°C using a tabletop centrifuge to pellet the nuclei and collect the post-nuclear supernatant (PNS) fraction. For subsequent centrifugation steps, samples were loaded into thin-walled ultracentrifuge tubes (Beckman Coulter 344090). The PNS fraction was then loaded onto a Nycodenz (Accurate Chemical and Scientific Corp 1002424) gradient with 110 µL of 22.5% Nycodenz on the bottom, 270 µL of 9.5% Nycodenz on the middle, and 225 µL of PNS on top. Samples were then subjected to centrifugation at 38,600 rpm in a SW55 swinging bucket rotor (Beckman Coulter 342194) for 1 h at 4°C. Fraction 5/6, which is located at the interface of the 22.5% and 9.5% Nycodenz gradients, was diluted 1.25× and loaded on top of a discontinuous Percoll gradient consisting of 110 µL of 22.5% Nycodenz on the bottom, 300 µL of 33% Percoll (SIGMA P4937) on the middle, and 200 µL of Fraction 5/6 on top. Samples were then subjected to centrifugation at 27,600 rpm in a SW55 rotor for 0.5 h at 4°C. For every 240 µL of F6 fraction collected, 168 µL of 60% (wt/vol) OptiPrep density gradient medium (Sigma D1556) was added. Lastly, 408 µL of the F6/Percoll sample was loaded on the bottom of the tube, overlaid with 75 µL of 30% OptiPrep, and 120 µL of sucrose homogenization buffer on top and centrifuged at 27,300 rpm for 0.5 h at 4°C. Volumes collected for each fraction, as depicted previously (13): F1, 50 µL; F2, 50 µL; F3, 80 µL; F4, 30 µL; F5/6, 120 µL; F5, 80 µL; F6/Percoll, 80 µL; F6, 40 µL. All fractions collected were pooled from multiple gradients within each independent experiment.

### Flotation assay

Nycodenz flotation was performed as previously described (43). Lipidation products ± 2.5 µM Lem26 variants (pre-incubated for 0.5–1 h) were mixed with 80% Nycodenz (prepared in SNH buffer: 50 mM Tris pH 8, 100 mM NaCl, and 1 mM MgCl_2_) at a 1:1 ratio to yield 300 µL of the sample in 40% Nycodenz in 5 × 41 mm ultracentrifuge tubes (Beckman Coulter 344090). The samples were overlayed with a 250 µL middle layer of 30% Nycodenz and a top 50 µL layer of ADP-ribosylation reaction buffer. Sample tubes were subjected to centrifugation at 48,000 rpm in a SW55 swinging bucket rotor (Beckman Coulter 342194) for 4 h at 4°C. The liposomes and lipidated proteins were collected from the top 80 µL of the gradient. Equal percentages of input and flotation fractions were subjected to SDS-PAGE, with percent recovery (flotation/input) calculated by normalizing the densitometry measurements with the total starting volumes for each fraction (ex. input densitometry value * [150 µL of reaction volume/5 µL of input loaded into SDS-PAGE gel]).

## Data Availability

All data are available in the main text or the supplemental material. Requests for materials should be directed to K.R.P. or C.R.R.

## SUPPLEMENTAL MATERIAL

Supplemental Material mbio.03595-25-s0001.pdf Figures S1-S7.

ASM does not own the copyrights to Supplemental Material that may be linked to, or accessed through, an article. The authors have granted ASM a non-exclusive, world-wide license to publish the Supplemental Material files. Please contact the corresponding author directly for reuse.
