# Supplementary material for: Legionella Lem26 functions as an ATG8-activated effector that inhibits host autophagy
Source: mBio. 2026 Feb 5;17(3):e03595-25. doi: 10.1128/mbio.03595-25 (PMC12977470; doi:10.1128/mbio.03595-25)

Figure S1. Loss of Lem26 in cells lacking known autophagy-inhibiting effectors does not affect intracellular replication.

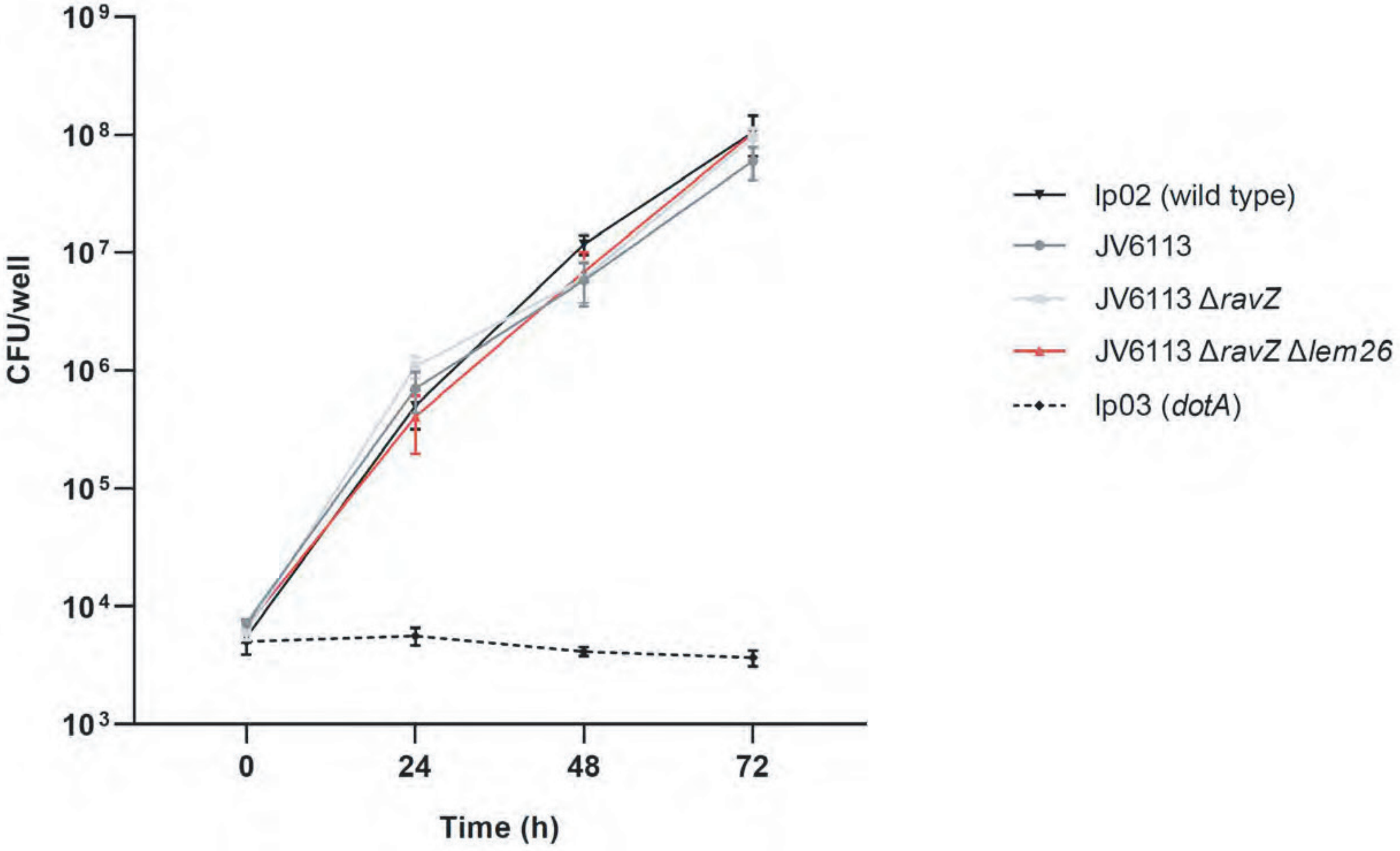

Figure S2. Lem26 does not inhibit the conjugation of Atg8 to single membranes (CASM) pathway induced by the TRPML1 agonist, ML-SA5.

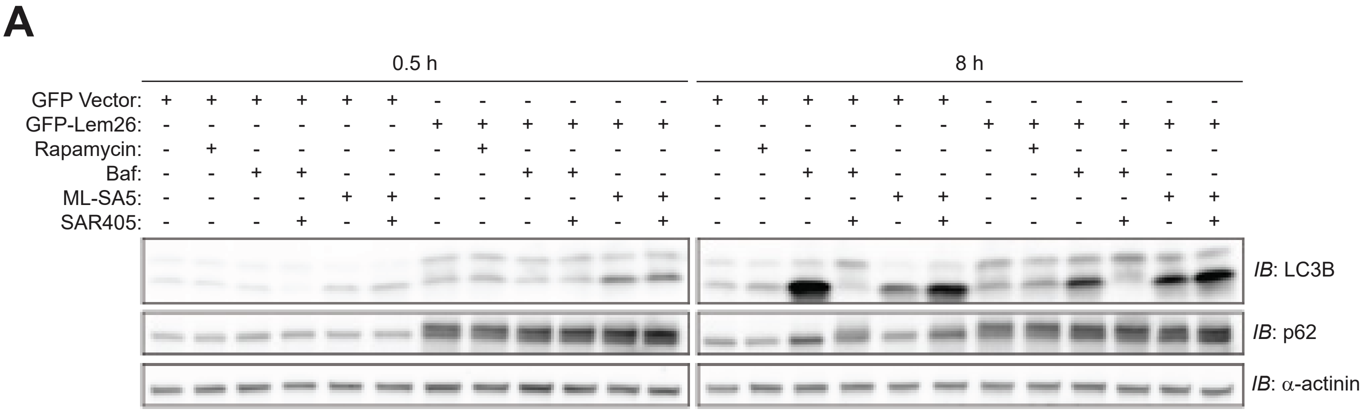

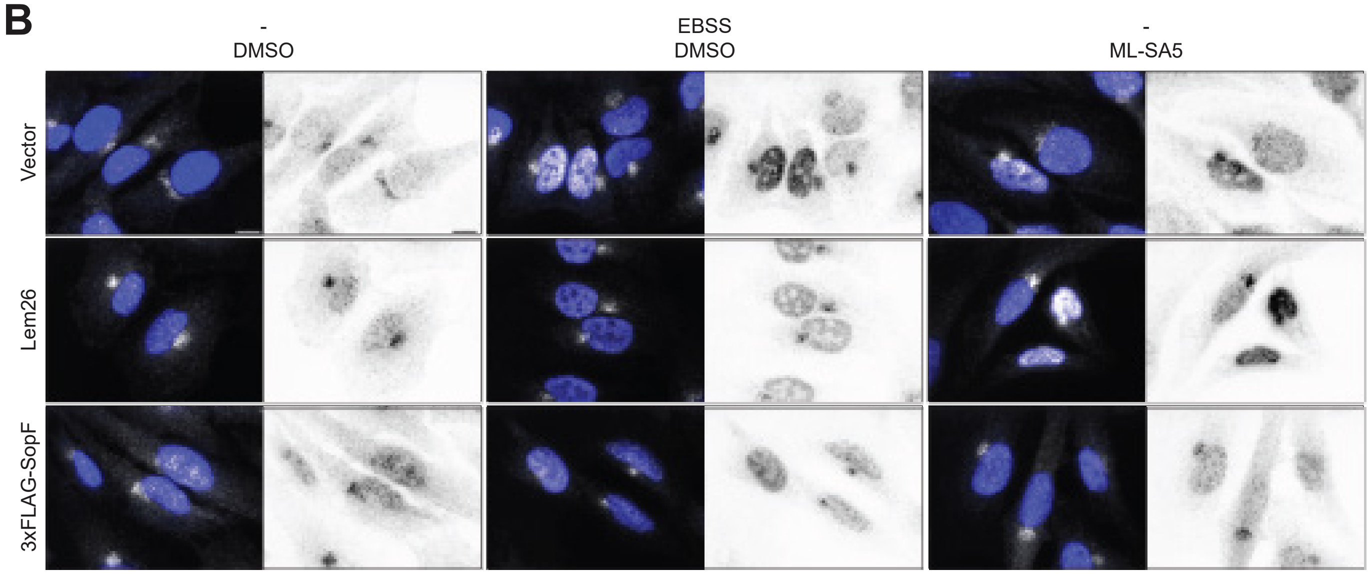

Figure S3. Lem26 does not inhibit the secretory and endosomal pathways.

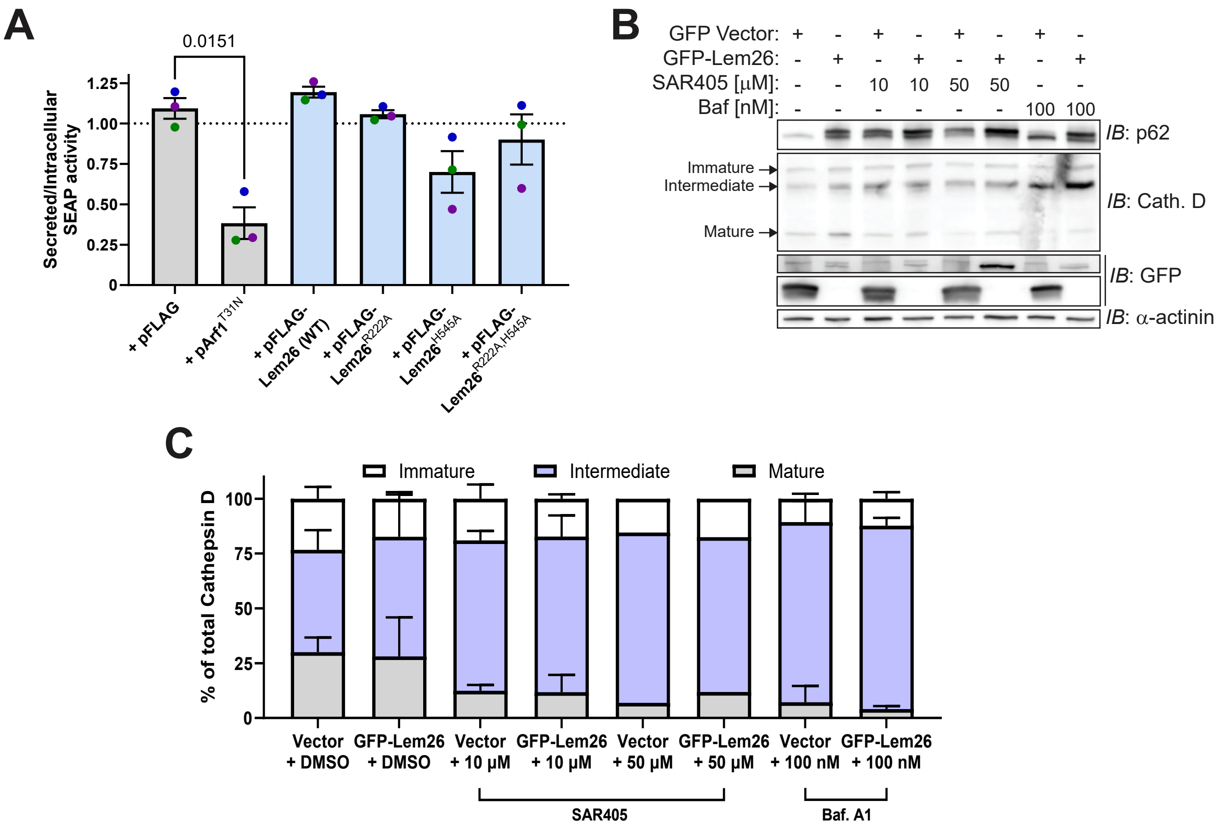

Figure S4. Several autophagy-related proteins are modified by Lem26 *in cellulo.*

A

	Protein Name	Lem26	$Lem26^{H545A}$	$Lem26^{R222A, H545A}$
		**Total Spectrum Count**		
1	ACTB	350	664	591
2	PLEC	296	500	450
3	KRT8	199	349	254
4	VIM	147	277	228
5	FLNA	109	262	222
6	KRT17	178	255	250
7	KRT7	122	215	174
8	SPTAN1	82	201	161
9	SPTBN1	86	195	174
10	KRT18	90	173	138
11	FLNB	65	158	136
12	ACTN4	41	149	90
13	DHX9	61	96	96
14	KRT19	46	85	61
15	ACTN1	20	84	49
16	HNRNPK	30	67	48
17	NPM1	45	64	61
18	TPM1	7	47	39
19	PLS3	17	46	35
20	FLNC	16	35	31
21	DSP	7	33	25
22	NCL	16	31	21
23	SQSTM1	23	30	19
24	ZC3HAV1	8	28	9
25	DBN1	10	27	22
26	SNRPD2	10	21	16
27	MTREX	9	20	12
28	FSCN1	11	19	6
29	AHNAK2	0	19	5
30	CTTN	3	18	12
31	PIK3R4	0	13	3
32	PARP14	10	12	0
33	EIF3E	5	9	4
34	RBM4	5	9	3
35	SVIL	0	9	2
36	Titin	7	9	0
37	PIK3C3	0	8	0
38	SIN3A	4	8	2
39	ITPR1	0	6	0
40	ERLIN2	5	6	2
41	ATG16L1	0	6	0
42	SPECC1	0	4	0
43	WDR76	2	4	0
44	CBX5	0	4	0
45	CDYL	0	4	0
46	SNX12	0	4	0
47	RPS26	0	4	0
48	PFKM	7	4	0
49	IGF2BP2	0	4	0
50	SREK1	0	3	0

B

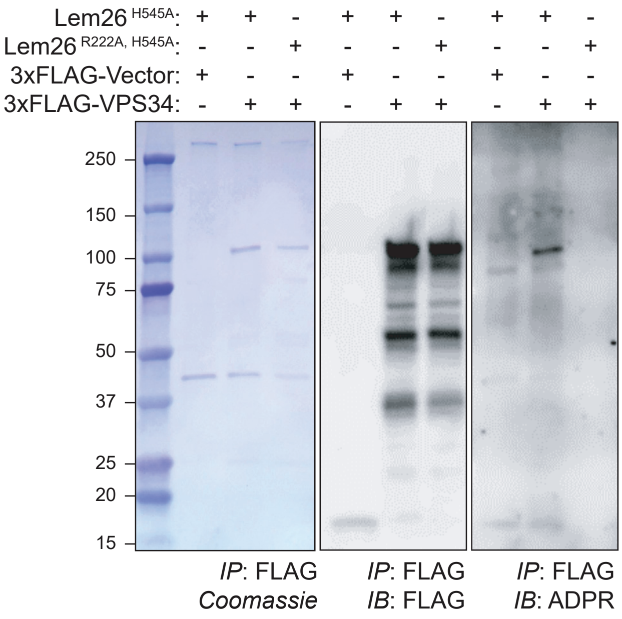

Figure S5. Membrane-conjugated GL1 promotes Lem26 membrane recruitment and enzymatic activity.

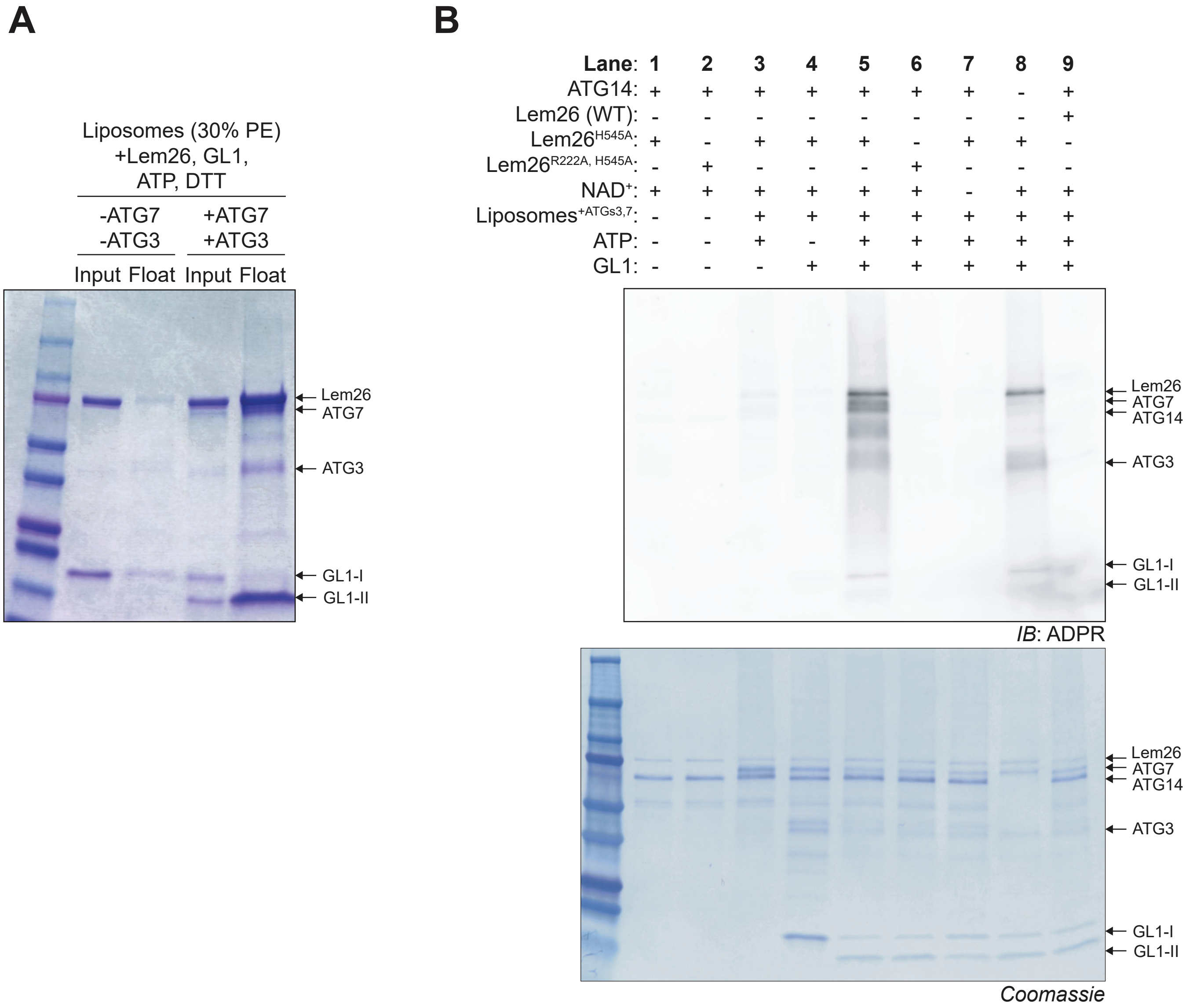

Figure S6. Lem26 possesses an Atg8-interacting motif (AIM) at its C-terminus.

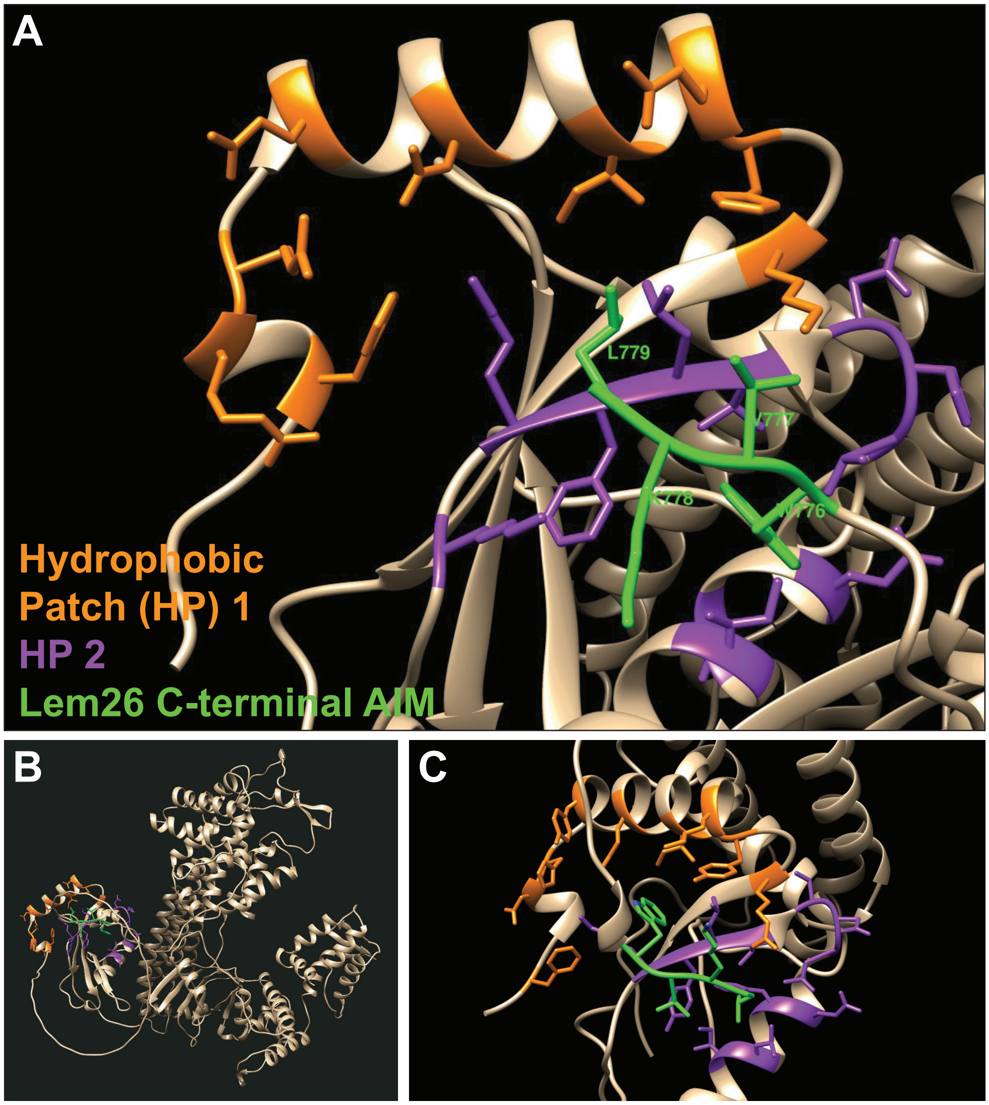

Figure S7. The Lem26 mART domain is activated by lipidated GL1 and PI3P.

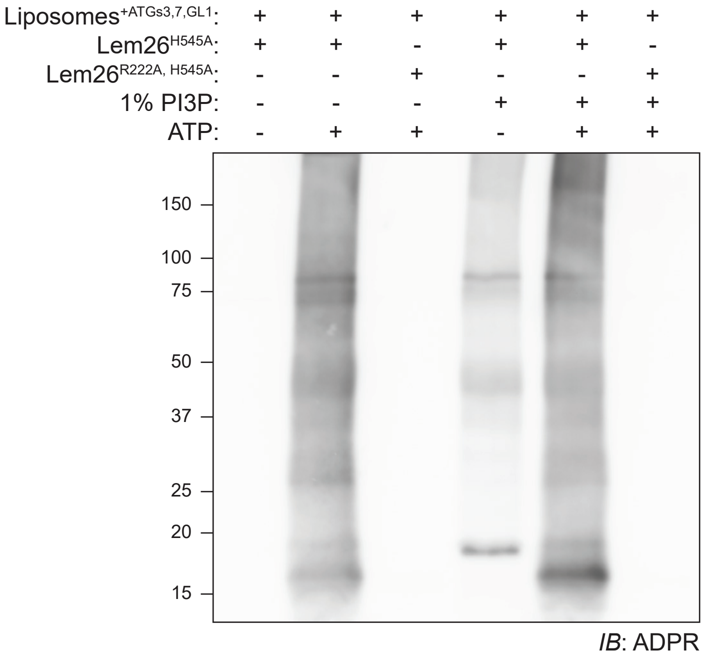

Supplement: Supplemental Material — Figures S1-S7. [file mbio.03595-25-s0001.pdf]
